# Outcomes of patients with hematologic malignancies admitted to the ICU

**DOI:** 10.1186/cc14619

**Published:** 2015-03-16

**Authors:** I Duayer, E Osawa, C Park, J Fukushima, J Almeida, R Nakamura, F Galas, L Hajjar

**Affiliations:** 1 Institute of Cancer of State of São Paulo, Brazil

## Introduction

In recent decades, therapeutic advances resulted in increased survival of patients with hematologic malignancies. These patients are increasingly admitted to the ICU due to infections, treatment toxicity and decompensation of chronic diseases. The aim of this study is to evaluate ICU, hospital and 6-month mortality in patients with hematological malignancies admitted to the ICU and to identify predictors of ICU mortality.

## Methods

We performed a retrospective study of 277 consecutive patients with hematological malignancies admitted to the ICU of the Institute of Cancer of State of São Paulo, Brazil, from January 2010 to December 2013. Patient clinical and laboratory characteristics, evaluation of organ dysfunctions and need for hemodialysis, mechanical ventilation and vasoactive agents in the ICU were collected. The primary outcome was ICU mortality. Data were analyzed with univariate and multivariate logistic regression.

## Results

The median age of the population was 57 years and 144 patients (52%) were male. Upon admission, 15 patients (5.4%) had disease remission and 31 (11.2%) had newly diagnosed disease. The ICU mortality rate was 26%, hospital mortality was 35.7% and 6-month mortality was 55.2%. The median number of organ dysfunction was 3 (IQR 2 to 4) and respiratory failure was the leading dysfunction, being present in 209 patients (75.5%). During the ICU stay, 21 patients needed hemodialysis (8%), 69 (25%) needed mechanical ventilation, 162 (58%) used vasoactive agents and 22 (8%) had a decision for limitation of medical treatment. On univariate analysis, risk factors for hospital mortality were acute myeloid leukemia, hospital stay prior to ICU admission >4 days, number of organ dysfunction ≥2, colonization and infection by a multidrug-resistant (MDR) agent, use of mechanical ventilation, use of vasoactive agents and renal replacement therapy. Multivariate analysis revealed that renal replacement therapy (OR = 6.35 (95% CI: 1.5 to 25.92), *P *= 0.010), SOFA score (OR = 1.69 (95% CI: 1.38 to 2.06), *P *< 0.001), RDW (OR = 1.27 (95% CI: 1.11 to 1.46), *P *= 0.001), lactate (OR = 1.04 (95% CI: 1.02 to 1.06), *P *< 0.001), colonization of MDR agent (OR = 10.73 (95% CI: 2.13 to 53.96), *P *= 0.004) and hospital stay prior to ICU admission >4 days (OR = 4.72 (95% CI: 1.8 to 12.3), *P *= 0.002) were predictive factors of ICU mortality.

## Conclusion

Patients from our institution have survival rates comparable with data from the literature. Our study suggests that mortality is associated with late ICU admission and colonization of MDR bacteria.

